# The contribution of moral injury to Israeli teachers’ mental health difficulties: the mediating role of shame and guilt

**DOI:** 10.3389/fpsyt.2025.1634795

**Published:** 2025-12-11

**Authors:** Nir Kaplan, Gadi Zerach, Yossi Levi-Belz

**Affiliations:** 1 Department of Clinical Psychology, Ruppin Academic Center, Emek Hefer, Israel; 2 Department of Psychology, Ariel University, Ariel, Israel; 3 School of Therapy, Counseling and Human Development, University of Haifa, The Lior Tsfaty Center for Suicide and Mental Pain Studies, University of Haifa, Haifa, Israel

**Keywords:** moral injury, teachers, burnout, depression, anxiety

## Abstract

**Introduction:**

Exposure to potentially morally injurious events (PMIEs) has been found to contribute to mental health difficulties (MHD). However, research on PMIE exposure and its consequences among teachers is scant. In this study, we aimed to narrow this gap by examining the associations between teachers’ exposure to PMIEs and measures of depression, anxiety, burnout, and intention to leave the profession. Furthermore, we examined the mediating role of expressions of moral injury (i.e., shame and guilt) in these associations.

**Method:**

A sample of 253 Israeli teachers (186 female, 73%) aged 23-66 (*M*_age_ = 44, *SD* = 10.36) completed validated self-report questionnaires assessing the study variables.

**Results:**

The findings demonstrated that exposure to PMIEs contributed significantly to depression, anxiety, burnout, and intention to leave the profession. Through structural equation model analysis, we found that expressions of moral injury mediated the association between PMIEs and MHD.

**Discussion:**

This study underscores the need to address moral injury among teachers as an essential factor for maintaining their mental health, as well as the overall sustainability of the educational system. Early screening and interventions are needed to identify and treat teachers at risk for MHD stemming from moral injury.

## 1 Introduction

In recent years, the potential impact of *moral injury* (MI) has been investigated. MI refers to “an act of transgression that creates dissonance and conflict because it violates assumptions and beliefs about right and wrong and personal goodness [ … ] resulting in psycho-bio-social impairment characterized by diminished opportunity for ‘life affirmation’” (1, p. 698). MI begins with exposure to potentially morally injurious events (PMIEs), defined as acts of omission or commission committed by oneself or by others or betrayal by a trusted other in a high-stakes situation (1, 2). Empirical evidence has revealed three dimensions of PMIE: Events perpetrated by oneself (PMIE-Self), witnessing events perpetrated by others (PMIE-Other), and events perpetrated by others perceived as betrayal (PMIE-Betrayal; 3).

Building on the original model (1), more recent formulations (4, 5) highlight the dynamic process through which PMIE exposure can develop into MI. According to this updated framework, exposure to PMIEs initiates a profound violation of moral beliefs that, when attributed to stable and internal flaws of the self, triggers maladaptive emotions such as shame and guilt. Williamson et al. (2) emphasized that these emotions are not merely secondary outcomes but constitute the central clinical mechanisms underlying MI. When these processes persist, they manifest in PTSD symptoms, demoralization, and even suicidal ideation and behavior (4, 6). Supporting this theoretical model, Plouffe et al. (7) found that the experience of MI is shaped less by exposure to PMIEs per se and more by the resulting shame and guilt, while other studies demonstrated that these emotions mediate the relationship between PMIE exposure and mental health difficulties (8, 9).

Most studies addressing the negative consequences of MI have focused on the military context (10). These consequences include heightened levels of depression and anxiety, as well as suicidal ideation and suicidal behavior (6, 8, 11). A recent study (12) revealed, that among UK treatment-seeking veterans, there were high levels of comorbidity between PMIE exposure and CPTSD, a disorder with an extensive range of symptoms in the areas of emotional regulation, relationships and self-perception, following prolonged exposure to traumatic experiences (13). Earlier studies found persisting symptoms related to MI among veterans of Afghanistan and Iraq Wars (14), as well as the Vietnam War (15).

However, MI can also impact mental health in other professions and contexts. Several studies have highlighted the prevalence of expressions of MI among law enforcement officers (16), child protection professionals (17), healthcare workers (18, 19), and refugees (20). Nevertheless, while research on MI among war veterans is extensive, studies in other areas remain limited. However, growing evidence has drawn attention to the presence of MI in various aspects of life (10).

Among the professional groups at risk, teachers may be especially vulnerable given the complex demands of their role. The 21^st^ century has posed teachers some of the most difficult challenges faced by the profession. Working for what teachers feel is inadequate compensation, lack of advancement opportunities, lack of administrative support, and dissatisfaction with working conditions are some of the challenges suggested by both researchers and teachers (21, 22).

These and other challenges teachers face have a high toll. Mental health difficulties (MHD) among teachers have become increasingly common, manifested in several domains, including high levels of burnout, stress, exhaustion, and depression and anxiety symptoms (23, 24). These difficulties have led inevitably to a rise in teachers considering leaving their profession. This trend appears to have reached its peak (or lowest point) following the COVID-19 pandemic (24). Another key motive cited by teachers deciding to leave the profession is a low sense of meaningfulness–teachers quit because they do not want to feel worthless (25).

For teachers, PMIEs may arise in diverse forms — from being exposed to or witnessing incidents of student abuse or neglect (26), to being pressured to act against their ethical or pedagogical principles (27), or even encountering large-scale traumatic events such as school shootings (28). Although these experiences differ in scope and severity, they all share a potential to undermine teachers’ moral integrity and contribute to distress, guilt, or shame.

Among the very few studies on the impact of MI on teachers, Currier et al. (29) found PTSD symptoms associated with PMIE exposure among teachers, and Sugrue (30) revealed a linkage between PMIE exposure and teachers’ intent to leave the profession. However, these studies addressing teachers focused primarily on events outside the school setting rather than within the education system (29) or suffered from a low response rate (30). Complementing these limitations, Oberg (27) provided qualitative evidence of structural and institutional dimensions of MI in teachers, pointing to areas that remain underexplored in quantitative research.

It is vital to investigate the prevalence of MI among teachers in different regions of the world, with a focus on the consequences of their PMIE exposure. These consequences can include mental (i.e., depression and anxiety) and practical (i.e., burnout and the intention to leave the teaching profession) outcomes.

### 1.1 The current study

Building on the growing body of research into the potential impact of MI on various professions, this study aims to explore its specific repercussions within the teaching profession. The present study aims to empirically examine the extent to which teachers are exposed to PMIEs and explore the psychological mechanisms contributing to the development of the noted mental health symptoms. To achieve this, we analyzed a broad sample of teachers in Israel in 2023. We aim to illuminate the role of MI in shaping these outcomes and contribute to the emerging body of literature on the impact of PMIEs on teachers’ mental health. Thus, we posit the following hypotheses:

- Hypothesis 1: Greater exposure to PMIEs among teachers is associated with higher levels of expressions of MI (i.e., shame and guilt), as well as higher levels of depression, anxiety, burnout and intention to leave the profession.
- Hypothesis 2: Expressions of MI (i.e., shame and guilt) mediate the association between exposure to PMIEs and MHD. Thus, the stronger the relationship between exposure to PMIEs and expressions of MI, the more teachers feel depression, anxiety, burnout and intention to leave the profession.

## 2 Materials and methods

### 2.1 Participants

As presented in **Table 1**, the sample comprised 253 Israeli teachers, including 73% females (*n =* 186) and 27% males (*n* = 68), with a mean age of 44 (*SD* = 10.36). Of the participants, 95% (*n* = 240) reported being Jewish, one participant reported being Christian, one Muslim, 10 atheists, and one reported “other.” Regarding religiosity status, 64.5% of the participants reported being secular, 19.5% moderately observant, 16% religious, and 1% orthodox. **Table 1** presents the participants’ demographic data.

**Table 1:** Demographics of the sample (n=253).

Demographic Value	Sub-groups	Frequency	Percentage	Range	Mean	SD
Age				23-66	44	10.36
Gender	Male	68	27			
	Female	186	73			
Religion	Jewish	240	95			
	Christian	1	4			
	Muslim	1				
	Atheist	10				
	Other	1				
Religiosity status	Secular	163	64.5			
	Moderately observant	49	19.5			
	Religious	40	16			
	Orthodox	1				
Profession	Class educator	150				
	Instructor	178				
	Headteacher/principal	70				
School type	State school	225	89			
	Religious school	20	8			
	Democratic school	1	3			
	Other	7				
Pupil class	Elementary	105	41.5			
	Junior high	52	20.5			
	High School	96	38			
Number of years teaching				Jan-40	14.84	10.64

Among the participants, 150 worked as homeroom teachers, 178 were instructors of specific subjects, and 70 were either headteachers or principals (some teachers held multiple roles in the school). The mean years of teaching was 14.84 (*SD* = 10.64). Of the participants, 225 worked in state schools, 20 in religious schools, one in a democratic school and seven in other types of schools. The sample included 41.5% (*n* = 105) who taught in elementary schools, 20.5% (*n* = 52) taught in junior high schools, and 38% (*n* = 96) taught in high schools.

### 2.2 Measures

#### 2.2.1 Potentially morally injurious events

To measure PMIEs, we used the Moral Injury Event Scale-Civilian questionnaire (MIES-C; 31). This 9-item self-report questionnaire taps exposure to perceived committed transgressions among civilians, comprising nine statements that participants rated on a 6-point Likert-type scale ranging from 1 (*strongly disagree*) to 6 (*strongly agree*). This scale was adapted from the Moral Injury Events Scale (MIES; 3), which was developed to assess MI within a military framework. The MIES-C adaptation included modifications to several original instructions and items to eliminate military references. For instance, the statement, “I feel betrayed by fellow service members whom I once trusted,” was revised to “I feel betrayed by friends whom I once trusted” (31). The MIES-C yields three subscales: (1) MIES-Self – four items describing PMIE exposure due to acts or decisions taken by the respondent, which they felt were morally wrong (e.g., “I am troubled by having acted in ways that violated my own morals or values”); (2) MIES-Other – two items describing PMIE exposure due to acts committed by others, which the respondent has witnessed, or about which the respondent has learned (e.g., “I am troubled by having witnessed others’ immoral acts”); and (3) – MIES-Betrayal – three items describing PMIE exposure due to perceived deceit or betrayal by others (e.g., “I feel betrayed by colleagues who I once trusted”). In line with Sugrue (30), we adapted the scale to the educational context. In the current sample, all three subscales yielded high internal consistency: MIES-Self (α = .88), MIES-Other (α = .85), and MIES-Betrayal (α = .79).

#### 2.2.2 Expressions of MI

To measure possible mental expressions due to PMIE exposure, i.e. shame and guilt, we used the Expression of Moral Injury Scale-Military-Short Form (EMIS-M-SF; 32), which we adapted to an educational context. The EMIS-M-SF comprises four statements (e.g., “I feel guilty about things that happened during my work as a teacher that cannot be excused”), presented to participants on a 5-point Likert-type scale ranging from 1 (*strongly disagree*) to 5 (*strongly agree*). Higher total scores indicate higher expressions of MI and higher severity. The EMIS-M-SF has been psychometrically validated in military samples [32]). Cronbach’s alpha coefficient for the current sample was high (α = .81).

#### 2.2.3 Depression

To measure depression, we used the Patient Health Questionnaire-2 (PHQ-2) (33). This 2-item questionnaire taps the core criteria for depressive disorders as delineated in DSM-5 (34) – “feeling down, depressed or hopeless” and “little interest or pleasure in doing things.” In the scale’s original version, the stem question preceding both statements is “Over the last 2 weeks, how often have you been bothered by the following problems;” As the questionnaire was distributed during the summer holiday, we modified it to fit the teachers’ experiences during the school year (“During the school year, how often did you feel…”). The items were presented on a 4-point Likert-type scale, ranging from 1 (*not at all)* to 4 (*nearly every day*). We used the total score, comprising the sum of both items (range of 2 to 8), as a continuous variable in our analyses. Higher scores reflect greater psychological distress. The PHQ-2 has been found to have good resemblance to the full PHQ-9 scale and has demonstrated high validity (33). Cronbach’s alpha coefficient for the current sample was.67.

#### 2.2.4 Anxiety

We used the Generalized Anxiety Disorder-2 scale (GAD-2; 35) to measure anxiety. Like the PHQ-2, the GAD-2 comprises two items introduced by the stem question, “Over the last 2 weeks, how often have you been bothered by the following problems?” As the questionnaire was distributed during the summer holiday, we modified the items to reflect teachers’ experiences during the school year (“During the school year, how often did you feel…”). The items were presented on a 4-point Likert-type scale ranging from 1 (*not at all*) to 4 (*nearly every day*). We used the total score, comprising the sum of both items (ranging from 2 to 8), as a continuous variable in our analyses. Higher scores reflect greater psychological distress. For the current sample, Cronbach’s alpha coefficient was.82.

#### 2.2.5 Burnout

We used the Copenhagen Burnout Inventory (CBI; 36) to measure the participants’ levels of burnout. The CBI is a 19-item self-report questionnaire that we adapted to teaching-related burnout (e.g., “Does it drain your energy to work with pupils?” (30)). The items were presented on a 5-point Likert-type scale ranging from 1 (*never or almost never/to a very low degree*) to 5 (*always/to a very high degree*). Cronbach’s alpha coefficient for the current study was high (α = .95).

#### 2.2.6 Intention to leave the profession

To measure the participants’ intention to leave the teaching profession, we used the self-report 4-item Intention to Leave Scale (ILS; 37). The participants’ answers to the first two items (“At this time in your career, would you want to quit your job if it were possible?” and “Are you actually planning to leave your job within the next 6 months?”) were rated as 0 = *no*, 1 = *not sure*, and 2 = *yes*. For the third item (“Are you actively searching for another job right now?”), response options were 0 = *no* and 1 = *yes*; for the last item (“Please indicate whether you have ever had thoughts of leaving your job”), response options were 1 = *never*, 2 = *occasionally*, and 3 = *frequently.*

#### 2.2.7 Open-ended item

In addition to the standardized measures, the questionnaire concluded with an open-ended item that invited participants to share any further reflections on their professional experiences and challenges as teachers. Responses to this item were subjected to an exploratory qualitative review, aimed at complementing the quantitative findings by identifying recurring themes and providing illustrative quotes.

### 2.3 Procedure

Data were collected between July 2023 and September 2023. Participants were recruited through WhatsApp and Facebook posts on several groups addressing Israeli teachers, as well as on the personal Facebook pages of the researchers and their acquaintances. Participants were provided with a link to an online version of the questionnaire, which was constructed using Qualtrics online survey software.

Upon accessing the questionnaire, all potential participants were informed of the risks and were assured of confidentiality, anonymity, and their right to withdraw from the study at any time. Participants were required to affirm their willingness to participate by signing an online informed consent form.

Following completion of the online questionnaire, gift vouchers were raffled and awarded to five participants. This study was approved by the ethics committee of the Ruppin Academic Center (Confirmation No. 220).

### 2.4 Data analysis

First, descriptive statistics of the participants’ demographic data were calculated, and relevant data regarding their experience as teachers were collected. In the second step, a series of Pearson correlation tests was performed to examine the relationships between the study variables. Next, three MANOVAs were conducted to examine differences between teachers who were exposed to PMIEs and those who were not with regard to depression, anxiety, burnout, and intention to leave the profession. Each MANOVA examined this in the three PMIE dimensions: PMIE-Self, PMIE-Other, and PMIE-Betrayal.

Next, we applied structural equation modeling (SEM) to assess our integrated mediation model of the contribution of expressions of MI on the relationships between PMIE exposure and the measured MHD. To obtain a full representation of the model, a combined rule of model fitness with the following accepted values was defined: a non-significant chi-square test (χ2), minimum discrepancy per degree of freedom (CMIN/DF) < 3 (38), normed fit index (NFI) > 0.95 (39), Tucker-Lewis index (TLI, also termed non-normed fit index [NNFI]; 40) > 0.95 (38), the comparative fit index (CFI) > 0.95 (41), and root mean square error of approximation (RMSEA) < 0.06 (42). The significance level for all statistical tests was set at.05. Analyses were conducted using the Statistical Package for the Social Sciences (SPSS, Version 25 for Windows) and AMOS software (Version 23).

## 3 Results

### 3.1 Prevalence of PMIEs among teachers

Descriptive statistics were calculated regarding the acts of transgression to which the participants were exposed. Aligning with Levi-Belz, Shemesh & Zerach (6), we calculated the percentage of participants who reported levels of *slightly agree* or higher.

**Figure 1:**
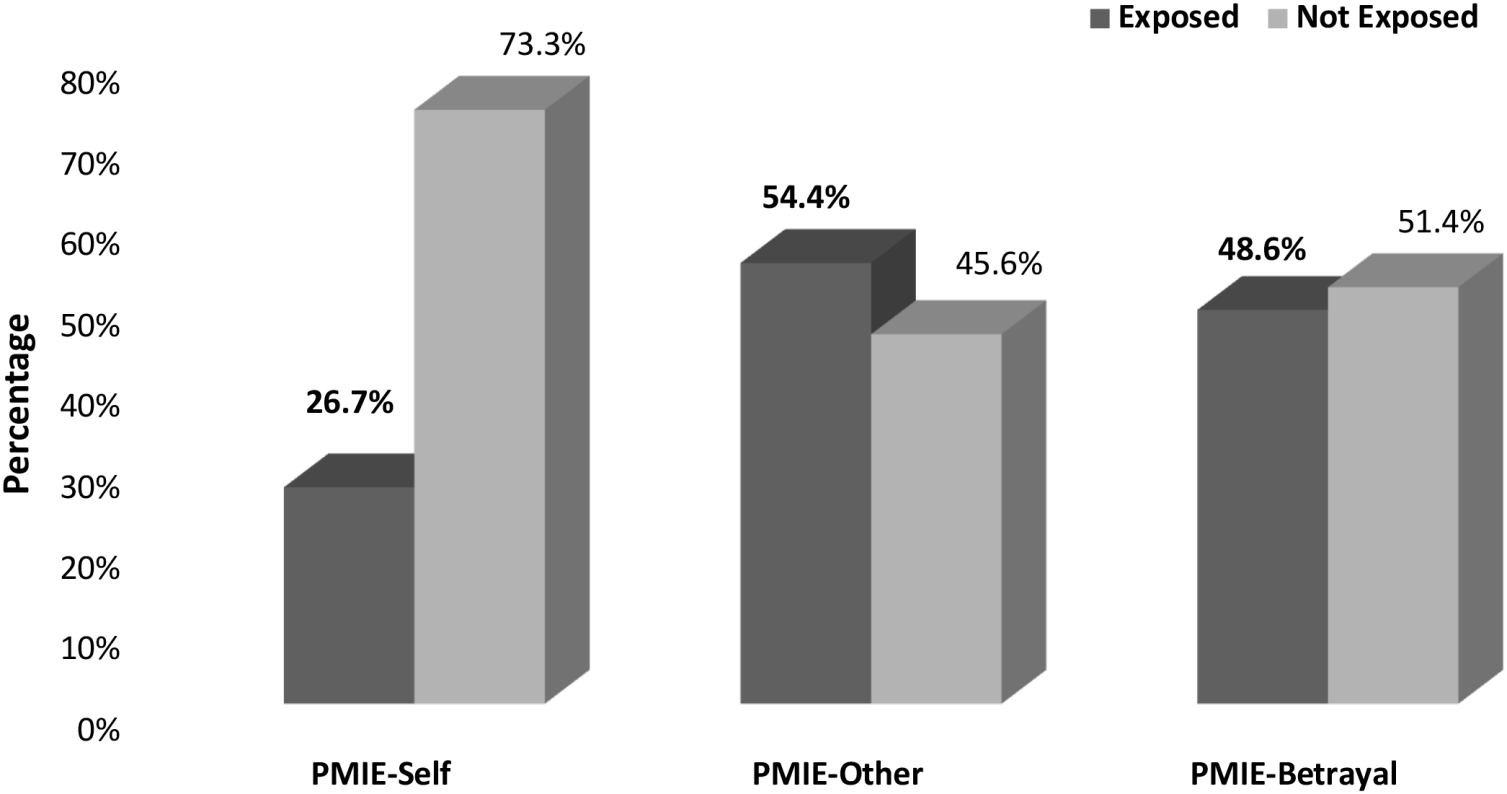
Prevalence of exposure to potentially morally injurious events (PMIEs) among teachers (n=253).

The most commonly endorsed items from the MIES were as follows: “I saw things that were morally wrong” (51.6%, *n* = 130, PMIE-Other), “I feel betrayed by figures of authority who I once trusted” (41.1%, *n* = 104, PMIE-Betrayal), and “I am troubled by having witnessed others’ immoral acts” (37.9%, *n* = 96, PMIE-Other). Overall, as presented in **Figure 1**, 26.7% (*n* = 67) of the participants endorsed at least one of the MIES-Self items, 54.4% (*n* = 137) endorsed at least one item from the MIES-Other subscale, and 48.6% (*n* = 123) endorsed at least one item from the MIES-Betrayal subscale.

### 3.2 Differences in MHD among teachers who were exposed vs. not exposed to PMIEs

To test the hypothesis that teachers exposed to PMIEs will experience more MHD, we performed a series of multivariate analyses of variance (MANOVA), with exposure to PMIE (Yes/No) as the independent variable. Depression, anxiety, burnout, and intention to leave the profession were the dependent variables. Age and the years of teaching were used as covariates. A MANOVA test was conducted for each of the three PMIE subscales. Aligning with Levi-Belz, Shemesh & Zerach (6), exposure to PMIE was defined as reporting a level of *slightly agree* or higher to at least one item of the relevant subscale.

#### 3.2.1 PMIE-Self

**Figure 2:**
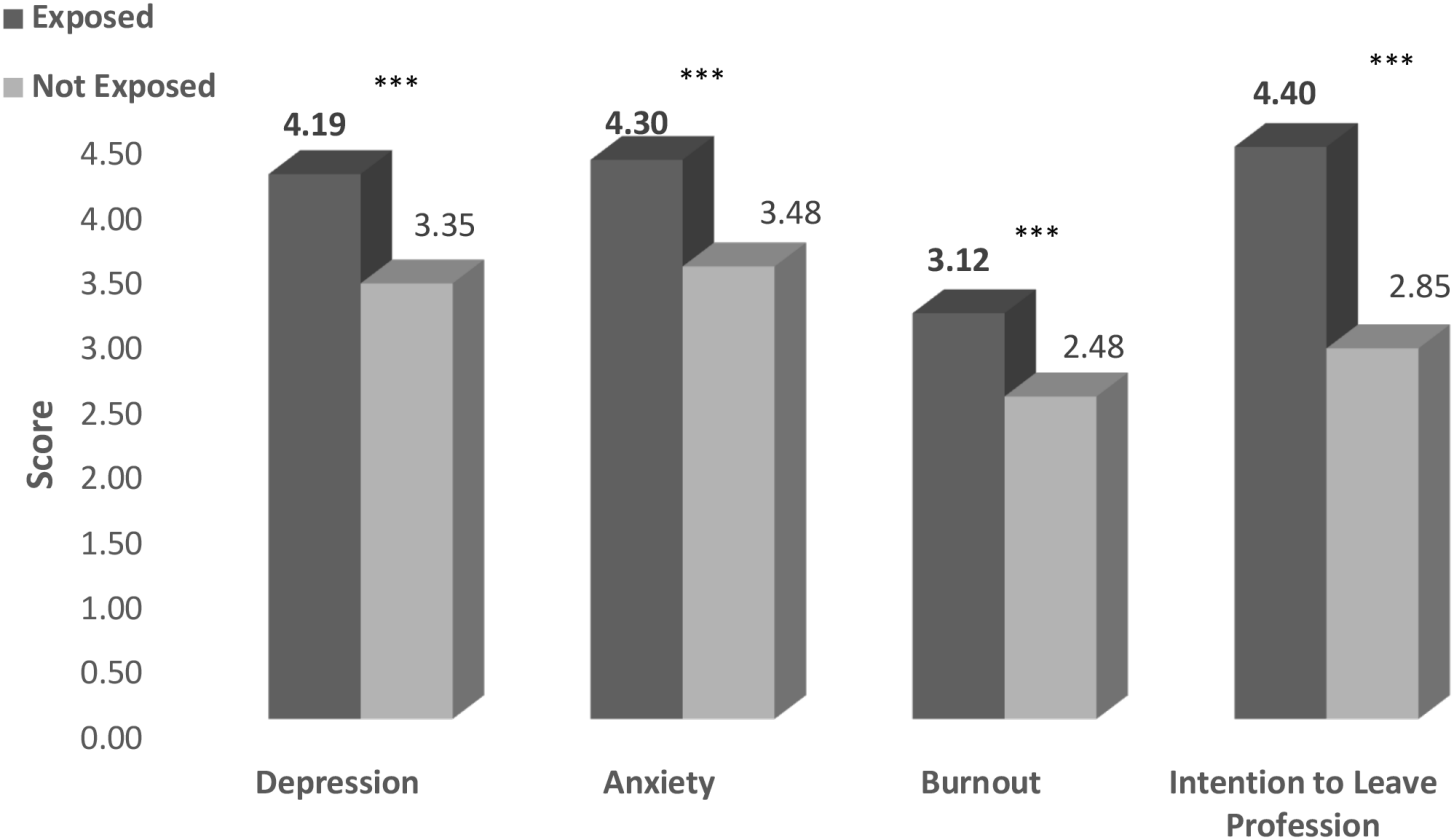
Levels of mental difficulties among teachers who were exposed to PMIE-Self and who were not exposed to PMIE-Self (n=253). *p < .05, **p < .01, ***p < .001.

The first MANCOVA revealed a group effect of all study variables between participants who endorsed at least one MIES-Self item and those who didn’t, Wilks’ *F* approximation (4, 242) = 11.267, *p* <.001, η^2^ = 0.157. As presented in **Figure 2** and in **Table 2**, the univariate ANOVA yielded a significant group effect for all four study variables, indicating that participants who endorsed at least one MIES-Self item reported higher levels of MHD than those who did not.

**Table 2:** Measures of mental difficulties among participants who endorsed at least one MIES-Self item and those who didn’t (n=253).

Measure		Exposed to PMIE-Self	Not Exposed to PMIE-Self	*F*	η^2^
		(n=67)	(n=186)		
Depression	*M*	4.19	3.35	17.56***	0.067
	*SD*	1.57	1.33		
Anxiety	*M*	4.30	3.48	14.17***	0.055
	*SD*	1.63	1.51		
Burnout	*M*	3.12	2.48	37.27***	0.132
	*SD*	0.74	0.74		
Intention to Leave the Profession	*M*	4.40	2.85	32.84***	0.118
	*SD*	2.14	1.80		

*p <.05, **p <.01, ***p <.001.

#### 3.2.2 PMIE-Other

**Figure 3:**
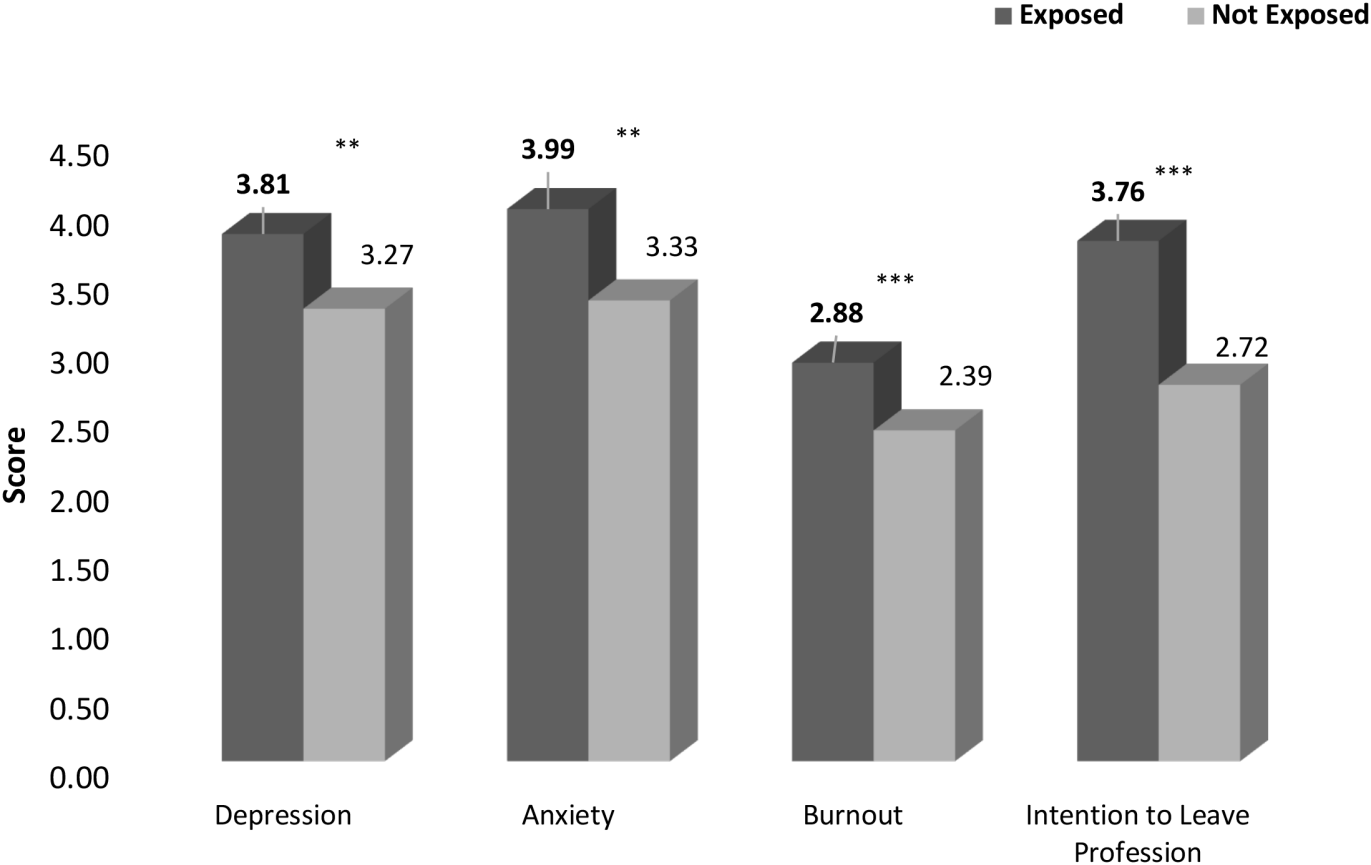
Levels of mental difficulties among teachers who were exposed to PMIE-Other and who were not exposed to PMIE-Other (n=253). *p < .05, **p < .01, ***p<.001

The second MANCOVA revealed a group effect for all study variables between participants who endorsed at least one MIES-Other item and those who did not (Wilks’ *F* approximation (4, 243) = 6.715, *p* <.001, η^2^ = 0.100). As presented in **Figure 3** and in **Table 3**, the univariate ANOVA yielded a significant group effect for all four study variables, indicating that participants who endorsed at least one MIES-Other item reported higher levels of MHD than those who did not.

**Table 3:** Measures of depression, anxiety, burnout and intention to leave the profession among participants who endorsed at least one MIES-Other item and those who didn’t (n=253).

Measure		Exposed to PMIE-Other	Not Exposed to PMIE-Other	*F*	η^2^
		(n=137)	(n=116)		
Depression	*M*	3.81	3.27	9.10**	0.036
	*SD*	1.47	1.34		
Anxiety	*M*	3.99	3.33	10.71**	0.042
	*SD*	1.61	1.47		
Burnout	*M*	2.88	2.39	24.62***	0.091
	*SD*	0.77	0.73		
Intention to Leave the Profession	*M*	3.76	2.72	16.99***	0.065
	*SD*	2.10	1.75		

*p <.05, **p <.01, ***p <.001.

#### 3.2.3 PMIE-Betrayal

**Figure 4:**
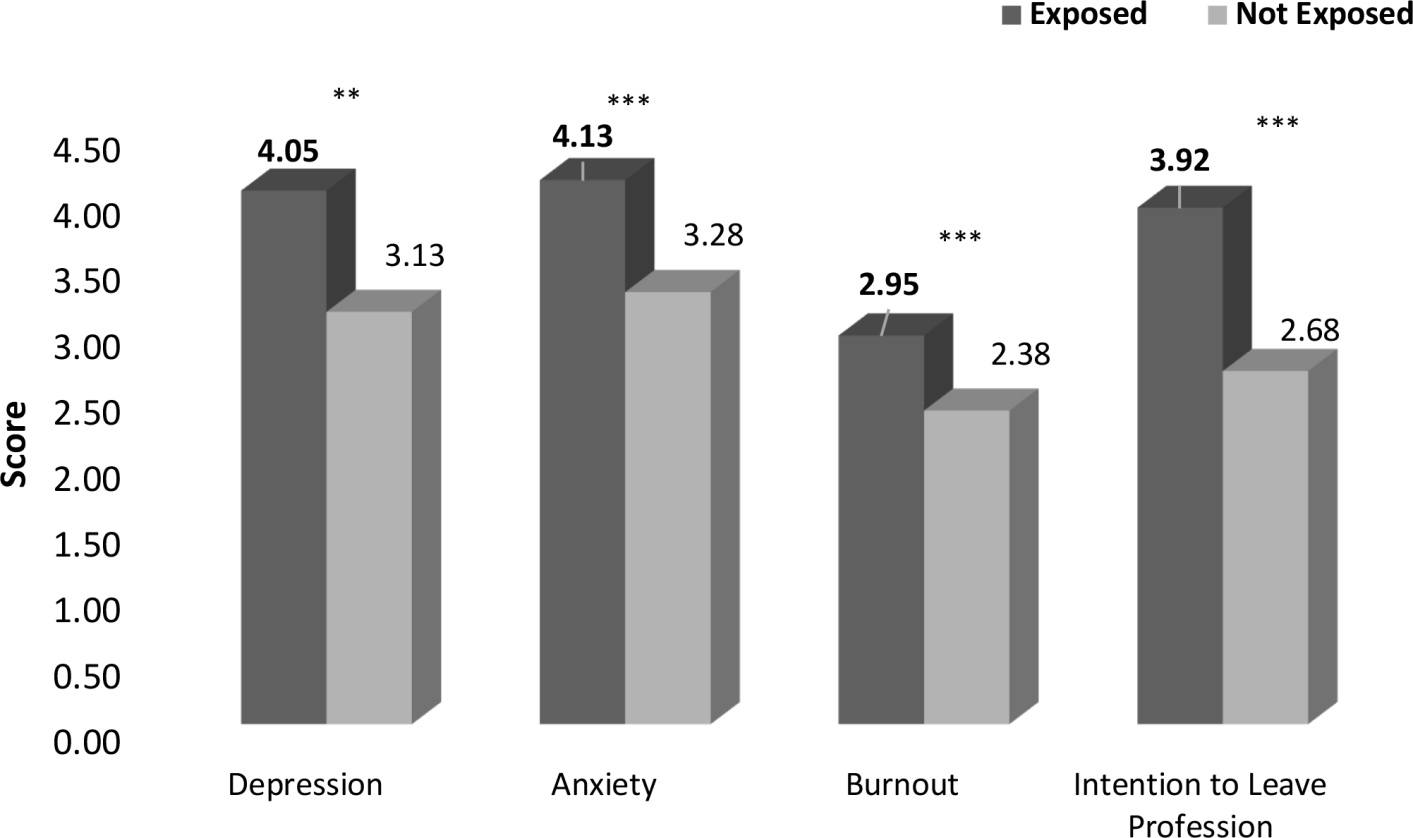
Levels of mental difficulties among teachers who were exposed to PMIE-Betrayal and who were not exposed to PMIE-Betrayal (n=253). *p < .05, **p < .01, ***p<.001.

The third MANCOVA revealed a group effect of all study variables between participants who endorsed at least one MIES-Betrayal item and those who did not, Wilks’ *F* approximation (4, 244) = 12.159, *p* < 0.001, η^2^ = 0.166. As presented in **Figure 4** and in **Table 4**, the univariate ANOVA yielded a significant group effect for all four study variables, indicating that participants endorsing at least one MIES-Betrayal item reported higher levels of MHD than those who did not.

**Table 4:** Measures of depression, anxiety, burnout and intention to leave the profession among participants who endorsed at least one MIES-Betrayal item and those who didn’t (n=253).

Measure		Exposed to PMIE-Betrayal	Not Exposed to PMIE-Betrayal	*F*	η^2^
		(n=123)	(n=130)		
Depression	*M*	4.05	3.13	27.45**	0.100
	*SD*	1.54	1.20		
Anxiety	*M*	4.13	3.28	20.81***	0.078
	*SD*	1.73	1.30		
Burnout	*M*	2.95	2.38	39.72***	0.139
	*SD*	0.72	0.75		
Intention to Leave the Profession	*M*	3.92	2.68	27.14***	0.099
	*SD*	2.00	1.84		

*p <.05, **p <.01, ***p <.001.

### 3.3 The contribution of PMIEs to expressions of moral injury, depression, anxiety, burnout, and intention to leave the profession among teachers

#### 3.3.1 Preliminary analysis

To examine the relationships between the different study variables, Pearson’s correlations were calculated and are presented in **Table 5**. First, the matrix shows significant positive correlations between PMIE exposure on all three subscales and expressions of MI, depression, anxiety, burnout, and intention to leave the profession. Moreover, expressions of MI were also positively correlated with depression, anxiety, burnout and intention to leave the profession. Age was significantly and negatively correlated with burnout but was not significantly correlated with any of the other variables, nor was years of teaching.

**Table 5:** Correlations between study variables (n=253).

	1	2	3	4	5	6	7	8	9	10
1 Age										
2 No. of Years Teaching	.76**									
3 PMIE – Self	-.03	.01								
4 PMIE – Other	-.02	.00	.49**							
5 PMIE – Betrayal	.05	.10	.42**	.64**						
6 Expressions of MI	.01	.04	.56**	.67**	.61**					
7 Depression	.04	.05	.31**	.30**	.43**	.40**				
8 Anxiety	-.10	-.03	.27**	.30**	.36**	.37**	.67**			
9 Burnout	-.14*	-.09	.40**	.37**	.45**	.41**	.53**	.54**		
10 Intention to Leave the Profession	-.08	-.06	.31**	.35**	.38**	.32**	.37**	.35**	.65**	
*M*	44.17	14.84	1.91	3.06	2.60	1.91	3.57	3.68	2.65	3.29
*SD*	1.36	1.64	1.10	1.50	1.33	.88	1.44	1.58	1.58	2.01
Range	23-66	1-40	1-5.75	1-6	1-6	1-4.75	2-8	2-8	1-4.79	1-8

*p <.05, **p <.01.

#### 3.3.2 Structural equation model

**Figure 5:**
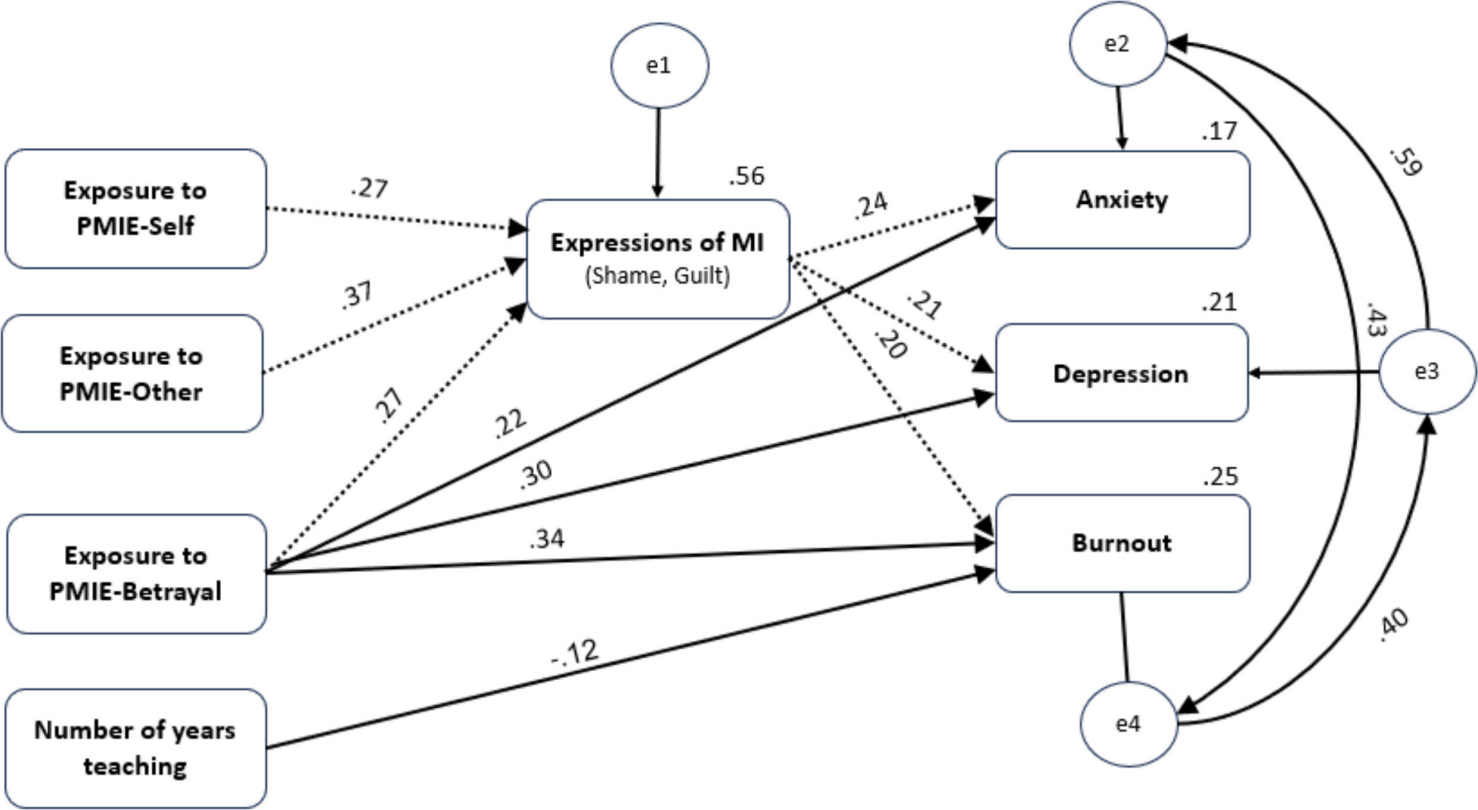
Serial mediation integrated model for anxiety, depression and burnout variables among teachers (n=253). Rectangles indicate measured variables. Number above or near endogenous variables represents the amount of variance explained (R^2^). Unidirectional arrows depict hypothesized directional links. Standardized maximum likelihood parameters are used. Direct paths are represented with solid arrows, indirect paths are represented with dashed arrows. All standardized paths were significant (*p* <.05 or lower). Only the PMIE-Betrayal dimension showed significant direct effects on the outcome variables, whereas PMIE-Self and PMIE-Other were related to these outcomes indirectly through Expressions of MI.

To more fully understand the mediation role of expressions of MI in the contribution of PMIE exposure and outcome measures of depression, anxiety, and burnout, a structural equation model (SEM) was employed. The chi-square goodness-of-fit index of the mediating model revealed an excellent fit with the data, χ2_(12)_ = 7.21; *p* = .12; CMIN/DF = 1.45; NFI = .98; TLI = .98; CFI = .99; RMSEA = .04. The statistically significant path coefficients are provided as standardized estimates in **Figure 5**.

As presented in **Figure 5** and in **Table 6**, exposure to PMIE-Betrayal contributed to mental health difficulties in two paths–both directly (depression – β = .30, anxiety – β = 21, burnout – β = .34) and indirectly through expressions of MI (depression – β = .05, anxiety - β = .06, burnout – β = .05). Exposure to the other two dimensions of PMIEs (PMIE-Self and PMIE-Other) was found to contribute only indirectly to depression (PMIE-Self – β = .06, PMIE-Other – β = .08), anxiety (PMIE-Self – β = .06, PMIE-Other – β = .09), and burnout (PMIE-Self – β = .05, PMIE-Other – β = .07) through expressions of MI. All the noted connections were found beyond the contribution of years of teaching.

**Table 6:** Direct and indirect standardized effects in the structural equation modeling analysis (n=253).

	Depression	Anxiety	Burnout
PMIE-Self			
Direct effect	.00	.00	.00
Indirect effect (through expressions of MI)	.06	.06	.05
Total	.06	.06	.05
PMIE-Other			
Direct effect	.00	.00	.00
Indirect effect (through expressions of MI)	.08	.09	.07
Total	.08	.09	.07
PMIE-Betrayal			
Direct effect	.30	.21	.34
Indirect effect (through expressions of MI)	.06	.06	.05
Total	.36	.28	.40

### 3.4 Exploratory qualitative findings

To complement the quantitative analyses, we conducted an exploratory review of teachers’ responses to the open-ended questionnaire item. One author reviewed all responses (n = 253) and inductively identified two main recurring themes that provide additional depth. The purpose of this process was to provide a contextual illustration of the quantitative results, rather than to conduct a full qualitative analysis. Below we summarize the main themes and illustrate them with representative quotations (translated from Hebrew).

#### 3.4.1 Leadership and institutional betrayal

Teachers frequently described a sense of disconnect between decision-makers and classroom realities, often framed as neglect or lack of support (e.g., “The highest frustration is the lack of appreciation for the teacher’s work and role by the state”, “Frustration comes from a salary that does not match the effort.”)

#### 3.4.2 Overload and emotional exhaustion

Participants also described overload and emotional exhaustion, framing these experiences not merely as workload issues but as a reflection of systemic neglect (e.g., “The workload is unbearable; it feels like we are constantly expected to do more with fewer resources”, “The supervisors demand tasks no one could possibly fulfill. By the end of the year, I had a breakdown.”)

## 4 Discussion

Exposure to potentially morally injurious events (PMIEs) contributes to various mental health difficulties (MHD), including depression and anxiety (43, 44). It also carries practical consequences such as burnout and the intention to leave one’s profession (45–47). However, although most existing research has focused on military populations (10), a growing body of evidence has revealed comparable outcomes in other professions and populations, such as first responders (48), healthcare and social workers (19, 43), and protesters (44).

In recent years, studies have begun to explore exposure to PMIEs and moral injury (MI) among teachers (29, 30). The current study aimed to expand upon this limited body of research by examining the prevalence of PMIEs and its contributions to MHD (depression and anxiety) in teachers, as well as practical consequences (burnout and the intention to leave the profession). In addition, we investigated the mediating effects that expressions of MI may have on these outcomes. To our knowledge, this is one of the first studies to address these questions and the first to empirically explore the contribution of teachers’ exposure to potentially morally injurious events (PMIEs) to their MHD.

The current findings underscore that PMIE exposure is indeed prevalent among teachers across all three subscales: Self, Other, and Betrayal. The results showed a compellingly high prevalence of PMIE exposure among teachers: 76.8% (*n* = 194) reported exposure to acts of transgression. Of the participants, 26.7% (*n* = 67) endorsed at least one item on the MIES-Self subscale, 54.4% (*n* = 137) on the MIES-Other subscale, and 48.6% (n = 123) on the MIES-Betrayal subscale. Interestingly, the MIES rates of the teachers in the sample are comparable to those found in military combatant settings (44) as well as those of veterans (6, 6, 8, 49).

Importantly, as hypothesized, PMIE exposure in all three dimensions was positively associated with depression, anxiety, burnout, and intention to leave the profession. It may thus be suggested that PMIE exposure contributes negatively to teachers’ mental and physical well-being and, consequently, may negatively affect the teacher-student relationship, the learning process, and even students’ psychological well-being (50, 51).

The study’s primary aim was to shed light on the possible mediators of the associations between PMIEs and MHD among teachers. As presented in previous studies with regard to other populations (52–54), this study demonstrated that MI symptoms of shame and guilt mediated the relationship between PMIE exposure and MHD. Interestingly, whereas shame and guilt mediated the effect across all three subscales in our study, the Betrayal subscale contributed to depression and anxiety levels, both directly and indirectly. These findings align with Litz et al.’s (1) conceptual model and are supported by evidence from various studies (e.g., 8, 49, 53), highlighting the deleterious effects of PMIE exposure. This exposure has thus been shown to lead to feelings of shame and guilt, which, in turn, exacerbate MHD, including levels of depression and anxiety. Notably, we also found that PMIE exposure in all three dimensions (Self, Other, and Betrayal) among teachers led to significantly higher levels of burnout and intention to leave the profession.

Litz et al. (1) emphasized the challenge of contextualizing or rationalizing one’s actions and those of others, especially the difficulty in integrating morally challenging experiences into one’s self-concept and worldview. This new and unsettling understanding could engender feelings of helplessness and disgust, which could naturally evolve into a sense of burnout (55). This trajectory may explain the patterns observed in the present study. Teachers entering the profession with a deep sense of purpose and belief in a just world are confronted with a system that includes figures and actions that they view as unjust and immoral, such as abuse of students, hypocrisy of principals and neglection of pedagogical objectives. The disillusionment stemming from this experience may accelerate burnout and, ultimately, foster a desire to leave the teaching profession altogether (56). Teachers’ narratives reinforced this pattern. For example, one participant wrote, “We entered teaching to make a difference, but today it feels meaningless.” These findings resonate with Oberg’s (27) work on MI in teachers, suggesting that ethical conflicts and professional disillusionment are often embedded within systemic dynamics, underscoring that MI among teachers cannot be fully understood without considering broader institutional and organizational contexts.

A central manifestation of these dynamics is the experience of betrayal, which emerged as a particularly salient theme in our findings, as 41.1% of the participants reported feeling betrayed by figures of authority they had once trusted. Overall, 48.6% of the participants reported experiencing betrayal resulting from PMIE exposure. This group exhibited significantly higher levels of depression, anxiety, burnout, and intention to leave the profession than those who did not experience betrayal following PMIE exposure. Unlike the other two PMIE dimensions (Self and Other), exposure to PMIE-Betrayal contributed to depression, anxiety, and burnout, both directly and through the mediation of feelings of shame and guilt. As one participant wrote, “The Ministry of Education has abandoned us, leaving teachers to deal with impossible situations alone”. Such reflections illustrate how feelings of betrayal may provoke frustration and anger beyond guilt and shame, consistent with prior conceptualizations (57, 58). Previous studies have shown that civilians and military personnel exhibit similar unique characteristics related to MI, particularly those derived from the Betrayal subscale (59).

This study has several limitations. First, data collection took place during the summer holiday and, thus, may not fully reflect teachers’ experiences during the school year. Additionally, the cross-sectional design of the study precludes drawing causal conclusions despite our application of a SEM analysis. A more heterogeneous sample may provide a fuller understanding of teachers’ experiences, as those not experiencing distress may have been underrepresented in this volunteer sample. For instance, a sample that includes additional demographics, such as Muslims and Orthodox Jews, may have provided a better representation of the Israeli population. As schools become more diverse, understanding how teachers from different backgrounds experience MI may reveal new insights into how to best support them.

Moreover, the use of self-reported questionnaires may have engendered response biases, including memory recall issues and social desirability. The study’s focus on Israeli teachers may have constrained its broader applicability, as the distinct sociocultural and geopolitical context may not be directly transferable to other populations. In addition, the exploratory qualitative component was conducted by a single reviewer and did not undergo inter-rater validation, which may have introduced subjectivity in the interpretation of the qualitative data. Lastly, whereas this study’s focus was on establishing the prevalence of MI among teachers, other variables that may have contributed to teachers’ mental health outcomes were not explored. Future research should investigate these factors, focusing on both teacher and PMIE characteristics that could either increase or reduce the risk of mental health issues. For example, teacher characteristics such as resilience, coping strategies, and perceived social support may act as protective factors. Similarly, PMIE characteristics like the frequency, severity, or perceived intentionality of the event, could influence the degree of psychological impact. Future research could also examine whether similar mechanisms linking institutional strain, MI and MHD operate in other professional context, as indicated in this study, as well as previous ones conducted among healthcare workers (19, 60).

Other directions for future research could include exploring the long-term effects of untreated MI on teachers, particularly regarding their ability to remain in their profession. It is crucial to investigate how PMIE exposure evolves longitudinally and how it may affect teachers’ mental health as well as their effectiveness in the classroom. Future studies could explore other potential mediating factors in the contribution of PMIE exposure to teacher distress. For instance, previous studies have revealed significant factors in various populations, including, but not limited to, thwarted belongingness (43) and the absence of self-disclosure (6).

### 4.1 Conclusions and implications

Addressing MI in teachers is crucial not only for their well-being but also for the overall health of the educational system. Teachers play a central role in shaping the experiences and learning outcomes of their students (61), making it essential to implement early screening measures to identify risks associated with teachers’ depression and anxiety (58). Many educators see it as their duty to serve as role models (62), which can lead them to underreport or fail to recognize their own conflicting emotions. The moral dimensions of their work further exacerbate this issue, as the expectation of maintaining a facade of strength might prevent them from seeking help (63). This internal struggle can result in unresolved guilt and shame, which contribute to the deterioration of mental health (58). Therefore, school leaders and mental health professionals should establish safe spaces for teachers to discuss their experiences openly. Without this transparency, the effects of school-related MI can persist, negatively impacting both educators and their students.

Once teachers are recognized as experiencing depression, it becomes vital to understand how MI can interfere with their treatment and support. The hidden nature of these emotions can lead to reluctance to seek help, with many educators fearing that their feelings of guilt or shame may undermine their professional authority and standing (64). This barrier to seeking assistance highlights the need for a supportive environment that normalizes these feelings. By creating interventions sensitive to the moral complexities of teaching, schools can foster a culture in which teachers feel safe expressing their emotions and accessing the care they need (5). Addressing these issues is critical for ensuring that teachers receive the support necessary to process their emotions and avert long-term psychological harm.

Interventions in the wake of MI must also focus particularly on the sense of betrayal (5), shown to be prominent in this study, highlighting the role of school leadership. Feelings of betrayal by authority figures can be especially damaging given the hierarchical nature of educational institutions (65). In other words, teachers rely on their leaders for guidance and support. When that trust is compromised, it can profoundly affect their professional identity (66). Consistent with this interpretation, Oberg (27) emphasizes that MI in teachers often arises not only from isolated incidents, but from systemic dynamics. This highlights the importance of designing interventions that address both individual experiences and broader organizational structures. Thus, training school leaders, such as principals and headteachers, to recognize signs of MI and respond with empathy and sensitivity is critical. By fostering a supportive leadership culture, schools can mitigate the damaging effects of betrayal and promote a healthier, more resilient teaching workforce, while helping principals work toward repairing trust within the institution.

Building on these findings, we propose a conceptual framework outlining potential intervention pathways across individual and systemic levels. At the individual level, reflective practice, moral-repair work, psychoeducation and mental-health support may assist teachers in processing MI. At the systemic level, interventions may include ethical leadership training, institutional acknowledgement of MI and policy development to prevent PMIE exposure. Future research should empirically examine these proposed pathways to determine which intervention strategies are most effective in preventing and addressing MI among teachers.

“There’s a feeling those in charge aren’t in touch with what actually happens in the schools,” “This is an important study; I fully hope it will bring about change.” These are just some of the responses teachers shared in their open-ended questionnaire responses. Most of all, participants thanked us for our interest in the teachers’ opinions and experiences. This study was the first of its kind in Israel, as it called attention to what appears to be a phenomenon that has a profound impact on teachers’ mental health.

Teacher well-being has long been researched in recognition of its importance to students’ well-being as well as to the effectiveness of the public educational system as a whole. As one participant in our research responded, “I see it as my mission and my destiny to teach the children of Israel. But,” this teacher added, “sometimes the burden is just too much.” This study has shown that there may be a simple way to begin unloading this burden. Perhaps all we need to do is what we have been taught since the first grade–we just have to listen to the teacher.

## Data Availability

The raw data supporting the conclusions of this article will be made available by the authors, without undue reservation.
